# GEAR Genomics: a user-friendly, open-source web platform enabling interactive genomic analysis for molecular biologists

**DOI:** 10.1093/nar/gkag445

**Published:** 2026-05-11

**Authors:** Andreas Untergasser, Markus Hsi-Yang Fritz, Vladimir Benes, Tobias Rausch

**Affiliations:** European Molecular Biology Laboratory (EMBL), Genomics Core Facility, Heidelberg 69117, Germany; European Molecular Biology Laboratory (EMBL), Genomics Core Facility, Heidelberg 69117, Germany; European Molecular Biology Laboratory (EMBL), Genomics Core Facility, Heidelberg 69117, Germany; European Molecular Biology Laboratory (EMBL), Genomics Core Facility, Heidelberg 69117, Germany

## Abstract

Many routine genomics tasks in molecular biology still depend on heterogeneous and proprietary software tools that hinder accessibility, reproducibility, and seamless laboratory use. We present GEAR (https://www.gear-genomics.com/), a unified, web-based genomics framework that provides a collection of lightweight, interactive applications for common molecular biology and genomics analyses directly in the browser. GEAR requires no software installation, user registration, or licensing and is designed for rapid, intuitive use without prior bioinformatics expertise. The platform integrates robust, well-established backend algorithms with modern web technologies to support a diverse set of tasks, including Sanger chromatogram visualization, alignment and variant detection, primer and padlock probe design, in-silico PCR, qPCR analysis, barcode generation and inspection, sequencing quality control, DNA manipulation, and sequence alignment visualization. In summary, GEAR serves as an integrated, open, extendible, and user-friendly genomics web server that consolidates a diverse set of tools within a single coherent framework with all code free and open-source (https://github.com/gear-genomics). By emphasizing interactivity, reproducibility, and ease of use, GEAR aims to support both routine laboratory tasks and exploratory genomic analyses across a broad range of research applications.

## Graphical Abstract

**Graphical Abstract:**
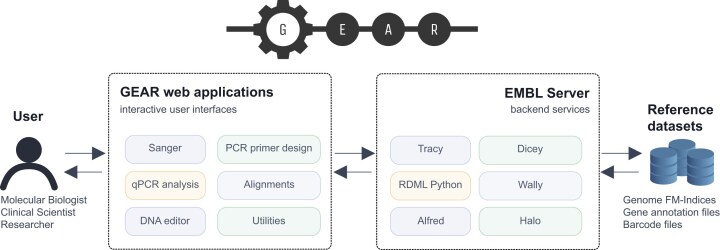

## Introduction

High-throughput and targeted sequencing technologies are indispensable in modern molecular biology, genetics, and genome research [1]. While large-scale pipelines and workflows exist for the automated processing of next-generation sequencing data [2], many routine and critical laboratory tasks, such as Sanger chromatogram analysis [3], qPCR primer and probe design [4], DNA sequence manipulation, barcoding, alignment, and quality control (QC), still rely on a heterogeneous collection of standalone desktop applications [5], command-line tools [6], online web services [7] and a plethora of commercial programs, which can compromise accessibility and integration into everyday laboratory workflows.

To address these challenges, we developed GEAR, a unified web-based framework that provides a collection of lightweight, interactive genomics applications accessible directly through a standard web browser. The primary goal of GEAR is to make common genomics analyses fast, intuitive, and reproducible, without requiring software installation, user registration, licensing fees, or prior computer science knowledge. By combining modern web technologies with well-established and thoroughly tested backend algorithms, GEAR bridges the gap between robust bioinformatics methods and their practical use in the lab.

## Methods and implementation

**Figure 1.**
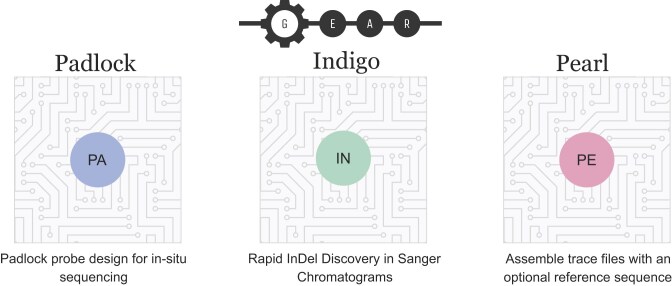
GEAR landing page: The modular GEAR web application framework provides a suite of tools as individual apps accessible from the landing page. Shown here are two apps for Sanger chromatogram analysis and assembly (Indigo and Pearl) and a tool for designing padlock probes for *in situ* sequencing.

The scientific applications of GEAR span tools for Sanger sequencing analysis, primer design, and *in-silico* PCR, probe design for *in-situ* sequencing, applications for quantitative PCR analysis, sequence barcode generation and verification, and sequence alignment and quality control. GEAR also supports genome alignment visualization, DNA manipulation, and sequence analysis (Fig. 1, Supplementary Table S1).

### GEAR genomics framework

The GEAR Genomics applications are built on a modular framework in which computational backend tools are accessed through lightweight web and visualization components, enabling researchers to interactively explore and analyze sequencing data. The suite is organized through a central landing page, providing a seamless interface to navigate the available apps (Fig. 1, Supplementary Table S1). Most apps share a clean, minimal layout designed for ease of use, with multiple tabs separating the main sections, built-in example data to illustrate functionality, and links to source code, documentation, and citation information. Each app is available under a stable URL (https://www.gear-genomics.com/<app>/), and all assets are delivered via the AWS content delivery network CloudFront, ensuring robust and rapid performance. Most apps follow a classical client-server design with a synchronous job model, providing immediate results. Because tasks are handled on demand without queuing, users can utilize all functionality without registration or providing an email, supporting fast, uninterrupted interaction with the data.

### Sanger trace visualization, assembly, and variant discovery

We developed four browser-based web applications for working with Sanger chromatogram traces, namely *Teal, Sage, Indigo*, and *Pearl*. Across all four tools, users upload standard Sanger trace formats (e.g. .abi/.ab1/.ab!/.ab and .scf) and (optionally) a reference genome through the client interface. The client submits the data to a server endpoint, which returns a JSON file to the client with the results. *Teal* provides an interactive chromatogram viewer that renders electropherogram peak traces (A/C/G/T) together with base calls and associated quality values, enabling rapid manual inspection. *Sage* extends this viewer to reference-aligned traces, displaying the aligned reference sequence alongside the chromatogram and reporting orientation (forward/reverse) and genomic coordinate context. *Indigo* focuses on SNV and InDel discovery in DNA samples, visualizing trace signals and basecalls and presenting alignment and variant summaries, with options to export results (e.g. variant downloads). *Pearl* validates DNA sequences, such as plasmids, using Sanger sequencing. Multiple sequences can be uploaded and assembled along with the expected reference sequence. A color-coded overview of matches and conflicts then displays all sequences in a multiple alignment, allowing the user to interactively analyze each conflict to verify or modify the called base for each conflict position. *Pearl* exports the curated sequence and the complete JSON format of the multiple alignment to ensure reproducibility. Relative to our earlier publication [8], the current implementations emphasize interactive, user-friendly web-based inspection and curation. In particular, the trace viewers now support modern in-browser usability features such as mouse-driven panning and drag navigation, wheel-based zooming, hover tooltips for quality inspection, selection-to-zoom behavior from the displayed sequence, and one-click copy and export of sequence ranges.

### Primer design and in-silico PCR

*Primer3Plus* is a well-known web interface running Primer3 on the server [9, 10], which we provide as a backend service. It can select amplification primers for regular or quantitative PCR (qPCR), as well as primers for Sanger sequencing. The user can control the primer selection using various parameters, making it suitable for many primer design applications.

*Silica* complements Primer3 by offering in-silico PCR. It locates the position of multiple primer sequences in a selected reference genome and calculates the melting temperature for each binding using the same algorithms as in Primer3. Furthermore, it checks whether the primers are close enough to each other to form an amplicon. Each binding candidate is linked to the UCSC Genome Browser for further inspection. *Silica* has two main applications: quickly checking the position of known primers in a genome and evaluating undesired secondary binding sites in newly developed assays.

### RDML-tools

The RDML-Tools analyze quantitative PCR data [11]. The software suite includes the analysis of amplification and melting curves, interplate correction, and statistical evaluation and visualization of experiments. Furthermore, the software implements geNorm for the identification of reference genes [12]. RDML-Tools supports various machine export formats and saves the data in the open RDML format [13].

### Genomic barcoding, distribution, and sequencing quality control

We also provide three web applications for common genomics workflows: sequencing QC (*alfred*), barcode design (*bgen*), and barcode inspection (*bistro*). All three applications are implemented as static HTML front ends with client-side JavaScript that drives interactive analysis and Plotly-based graphics. *Bistro* allows users to paste multiple DNA sequences (e.g. barcodes), compute per-position base frequencies, and render an interactive stacked bar chart of the base distribution across positions. *Bgen* focuses on barcode generation: it can start from either user-provided candidate barcodes or build candidates using a user-defined barcode length and amount. The tool then searches for a barcode set that maximizes pairwise dissimilarity (Hamming distance) while keeping per-position base composition balanced, enabling practical selection of robust barcode sets. *Alfred* [14] is a rich QC visualization frontend that uses the statistics file produced by *Alfred’s* command-line application as input: users upload one or more JSON reports, the app merges inputs, supports sample and read-group selection, and provides interactive Plotly charts and QC tables. QC summary tables can be exported as CSV files.

### DNA sequence alignment, visualization, and editing

The *Wily-DNA-Editor* is a plasmid editor written in HTML and JavaScript running entirely in the browser. It allows editing DNA sequences, finding restriction sites, simulating agarose gels, drawing plasmid maps, and translating open reading frames. The editor can handle features present in the GenBank file and curate a local library that can be used to find features in unannotated sequences. The modified sequences can be downloaded as a GenBank file, and all graphics can be saved as SVG files.

*Halo* targets haplotype-resolved sequencing alignment datasets. It offers interactive exploration of the data by sample or by chromosome, synchronized multi-panel viewing, region selection controls, and linked axes behavior to make comparative inspection easier. The method is suitable for visualizing structural variants in haplotype-resolved Strand-Seq [15] data where the DNA strand (‘Watson’ or ‘Crick’) reflects the parental haplotype.

*Maze* and *sabre* are two sequence alignment browsers. *Maze* generates so-called dotplot alignments of two input sequences using exact matches, and *sabre* is a multiple-sequence alignment browser where the input is a FASTA multi-align file. These two alignment viewers are complemented by *Salt* for local alignment computation using the Smith-Waterman algorithm.

Finally, *Wally* provides a simple web frontend for generating visualizations of short-read or long-read alignments via the *wally* CLI application [16]. The user submits a region and sample(s) to the backend API, which then returns a rendered alignment plot, with quick controls to zoom and shift the viewed window, and a time-limited PNG link for sharing and download.

### Probe design for in-situ sequencing applications

*Padlock* implements a padlock-probe design algorithm for in-situ sequencing applications [17] that generates candidate probes from user-specified targets provided as Ensembl gene/transcript identifiers or custom FASTA sequences. For reference-based targets, the tool extracts the requested genomic features (default: exons; configurable to CDS/UTRs/genes/transcripts) from a GTF/GFF3 annotation file, retrieves the corresponding reference sequence, and enumerates candidate padlock probe windows consisting of two arms of fixed length (default 20bp each) flanking a ligation junction. For each candidate, it applies sequence-quality constraints based on GC content bounds, thermodynamic filtering using Primer3’s thermodynamic model to ensure suitable melting temperatures, and balanced melting temperatures for each designed probe arm. The filtering of candidate probes also involves genome-wide specificity checks using an FM-index of the reference genome: by default, both arms must be unique, or alternatively, only one arm must be unique. To control off-target ligation, the method can additionally evaluate near-match neighborhoods of each arm via configurable Hamming or edit distances. Probe candidates with a large number of genomic neighbor hits are then discarded. Finally, accepted candidates are assembled into full padlock sequences by concatenating reverse-complemented arms with user-defined spacer(s), a fixed anchor sequence, and per-target (per-gene) barcode sequences (predefined or uploaded). Results are exported as a tab-delimited file with per-probe metrics and UCSC links for rapid manual probe inspection.

### Backend services

*Tracy* is a C++ command-line tool for the analysis of Sanger sequencing chromatograms [8]. It performs basecalling and trace alignment using a position-wise trace profile representation. Trace profiles are aligned using dynamic programming (semi-global or global depending on the task) with a DNA scoring scheme for matches, mismatches, gap-open, and gap-extend. For rapid mapping of a trace to an entire reference genome, *tracy* extracts a candidate reference slice using a pre-built FM-index of the reference genome. *Dicey* performs in-silico primer sequence validation by searching candidate oligonucleotides against a FM-indexed reference genome and applying thermodynamic filtering [9]. For primer search, primer pairs provided in FASTA are queried against the indexed genome to enumerate genomic binding sites (including off-targets) and to report predicted amplicons, including predicted melting temperatures. For padlock probe design, target regions are derived from gene models (GTF) and scanned to select two arms flanking a target sequence. Probe candidates are filtered by genomic uniqueness, GC-content, and thermodynamic properties. *Alfred* [14], *wally* [16], and *halo* are command-line applications for working with BAM alignment files. For the web applications with the same name, only the *qc* subcommand of *alfred* and the *region* subcommand of *wally* are used. *Halo* is a dedicated method to generate the input JSON file for the web application.

All of the above backend services are versioned and released on GitHub as a static binary, Singularity SIF file, and Docker container for reproducibility. Reference genomes and annotation files are hosted on the backend web server at EMBL.

## Web server accessibility

The GEAR web server is designed to be accessible and secure: It requires no user registration or email collection and stores data for a maximum of 24 h. Backend services run on a secure internal web server with HTTPS only. Result URLs use random universally unique identifiers (UUIDs), and there is absolutely no long-term user data storage. Users can explore all tools instantly with one-click example data, supported by detailed help pages. Outputs are interactive, downloadable, and linked to external resources (e.g. UCSC), which provides additional value. The website is free and open to all users, and there is no login requirement.

## Supplementary Material

gkag445_Supplemental_File

## Data Availability

All software code developed in this study is free and open-source (https://www.gear-genomics.com/licenses/), with public access provided through GitHub (https://github.com/gear-genomics) and Zenodo (https://doi.org/10.5281/zenodo.19705066).
